# Supplementary material for: Preeclampsia in pregnant women with COVID-19: a prospective cohort study from two tertiary hospitals in Southern Brazil
Source: PeerJ. 2024 Jun 11;12:e17481. doi: 10.7717/peerj.17481 (PMC11177852; doi:10.7717/peerj.17481)
Supplement: Supplemental Information 1 [file peerj-12-17481-s001.docx]

**CATEGORICAL DATA CODEBOOK**

| **VARIABLES** | **CODES** | |
| --- | --- | --- |
| PREECLAMPSIA | 0 = NO | 1 = YES |
| MATERNAL AGE | YEARS |  |
| MATERNAL AGE RISK | ≤ 34 YEARS OLD | > 34 YEARS OLD |
| NULLIPARITY | 1 = NO (Multiparity) | 0 = YES |
| HIGH RISK PRENATAL CARE | 0 = NO | 1 = YES |
| CHRONIC HYPERTENSION | 0 = NO | 1 = YES |
| OBESITY | 0 = NO | 1 = YES |
| CHRONIC HYPERTENSION AND OBESITY | 0 = NO | 1 = YES |
| GESTATIONAL DIABETES | 0 = NO | 1 = YES |
| PROM | 0 = NO | 1 = YES |
| COMORBIDITIES (OTHERS) | 0 = NO | 1 = YES |
| SARS | 0 = NO | 1 = YES |
| COVID-19 SEVERITY | MILD OR MODERATE ILLNESS WITHOUT ICU ADMISSION | SERIOUS ILLNESS WITH ICU ADMISSION |
| ACUTE COVID-19 AT THE TIME OF DELIVERY | 0 = NO | 1 = YES |
| CESAREUM DELIVERY | 0 = NO | 1 = YES |
| POSTPARTUM HEMORRHAGE | 0 = NO | 1 = YES |
| FETAL GROWTH RESTRICTION | 0 = NO | 1 = YES |
| FETAL DEATH | 0 = NO | 1 = YES |
| PLACENTAL HISTOLOGICAL CHANGES^A^ | 0 = NO | 1 = YES |
| PREMATURITY | 0 = NO | 1 = YES |
| BIRTH WEIGHT | GRAMS |  |
| LOW BIRTH WEIGHT | > 2.500G | < 2.500G |
| 5TH MINUTE APGAR SCORE | SCORE |  |
| APGAR 5TH MINUTE < 7 | 0 = NO | 1 = YES |
| NICU ADMISSION | 0 = NO | 1 = YES |
| ICU ADMISSION | 0 = NO | 1 = YES |
| TIME OF SYMPTOMS | DAYS |  |
| ICU LENGTH OF STAY (DAYS) > 7 | DAYS |  |
| MECHANICAL VENTILATION | 0 = NO | 1 = YES |
| PLASMAPHERESIS | 0 = NO | 1 = YES |
| PRONATION | 0 = NO | 1 = YES |
| CORTICOID THERAPY | 0 = NO | 1 = YES |
| TIME OF CORTICOTHERAPY > 10 | 0 = NO | 1 = YES |
| MATERNAL DEATH | 0 = NO | 1 = YES |
