# Supplementary material for: Preeclampsia in pregnant women with COVID-19: a prospective cohort study from two tertiary hospitals in Southern Brazil
Source: PeerJ. 2024 Jun 11;12:e17481. doi: 10.7717/peerj.17481 (PMC11177852; doi:10.7717/peerj.17481)
Supplement: Supplemental Information 3 [file peerj-12-17481-s003.doc]

STROBE Statement—Checklist of items that should be included in reports of ***cohort studies***

|  | Item No | Recommendation |  |  |  | Pages |  |  |  |  |
| --- | --- | --- | --- | --- | --- | --- | --- | --- | --- | --- |
| **Title and abstract** | 1 | (*a*) **Preeclampsia in pregnant women with COVID-19: A prospective cohort study from two tertiary hospitals in Southern Brazil** |  |  |  | 1 |  |  |  |  |
|  |  | (*b*) **Prospective cohort study of 100 hospitalized pregnant women from two tertiary hospitals, diagnosed with COVID-19, and divided into 2 groups: PE+ group (pregnant women with COVID-19 with preeclampsia) and PE- group (pregnant women with COVID-19 without preeclampsia).** **These pregnant women had prevalence, risk factors and maternal and perinatal outcomes data compared.** |  |  |  | 4 |  |  |  |  |
| Introduction | | |  |  |  |  |  |  |  |  |
| Background/rationale | 2 | COVID-19 is an infectious pathology that shows vascular changes during pregnancy, as well as placentas. |  |  |  | 2 |  |  |  |  |
| Objectives | 3 | To estimate the prevalence of preeclampsia and to study the risk factors for preeclampsia and maternal and perinatal outcomes in hospitalized pregnant women with COVID-19 with preeclampsia compared without preeclampsia. The main hypotheses were an increase in the prevalence of Preeclampsia in pregnant woman with severe COVID-19, as well as more unfavorable maternal and perinatal outcomes. |  |  |  | 4 |  |  |  |  |
| Methods | | |  |  |  |  |  |  |  |  |
| Study design | 4 | Prospective cohort study of 100 hospitalized pregnant women from two tertiary hospitals, diagnosed with COVID-19, and divided into 2 groups: PE+ group (pregnant women with COVID-19 with preeclampsia) and PE- group (pregnant women with COVID-19 without preeclampsia). These pregnant women had prevalence, risk factors and maternal and perinatal outcomes data compared. |  |  |  | 4 |  |  |  |  |
|  |  |  |  |  |  |  |  |  |  |  |
| Setting | 5 | **The research consists in an observational, prospective study with cohort design of 100 hospitalized pregnant women diagnosed with COVID-19 and divided into 2 groups: PE+ (with PE) and PE- (without PE). The study was conducted from July 2020 to July 2021, in two tertiary hospitals from Curitiba, in southern Brazil.** |  |  |  | **4** |  |  | **44** |  |
| Participants | 6 | (*a*) During the research, the data collection tools used were the software Tasy, the Management Application for University Hospitals, and the Hospital Information System. Microsoft Excel spreadsheets were used to record the data. The variables studied included: a) Gestational history: age of the pregnant woman (risk age greater than 34 years: absent or present), parity, usual or high-risk prenatal care; as main comorbidities studied: chronic arterial hypertension, obesity and gestational diabetes; b) Characteristics of COVID-19: tests performed for COVID-19, severity of respiratory symptoms, chest axial computerized tomography (chest CAT) and markers of inflammatory activity; c) Perinatal outcomes: type of delivery, postpartum hemorrhage (PPH), premature rupture of membranes (PROM), histological placental changes, fetal death, prematurity, low birth weight, 5^th^ minute Apgar score, need for hospitalization of the infant newborn in the Neonatal Intensive Care Unit (NICU). In the interpretation of perinatal outcomes, the criteria of the Brazilian Society of Pediatrics; d) Maternal outcomes: ICU admission, duration of COVID-19 symptoms, length of stay in the ICU, need for ventilatory support, plasmapheresis, pronation, maternal corticosteroid therapy and maternal death. The classification of COVID-19 was standardized according to the Brazilian Ministry of Health's Manual of Recommendations for Assistance to Pregnant and Postpartum Women Facing the COVID-19 Pandemic. Initially, 142 pregnant women with clinical suspicion of COVID-19, who were admitted from two tertiary hospital complexes, were studied. Of these, 42 were excluded by the criteria. Therefore 100 pregnant women with COVID-19 were included in the study |  |  |  |  |  |  | 55 |  |
|  |  | (*b*) Initially, 142 pregnant women with clinical suspicion of COVID-19, who were admitted from two tertiary hospital complexes, were studied. Of these, 42 were excluded by the criteria. Therefore 100 pregnant women with COVID-19 were included in the study. The PE+ group included 11 pregnant women and the PE- group included 89 pregnant women. |  |  |  |  |  |  | 6 |  |
| Variables | 7 | **Inclusion criteria**: hospitalized pregnant women with positive tests for SARS-CoV-2, through nasopharyngeal real time polymerase chain reaction (RT-PCR), rapid antigen test, and IgM and IgG serology; with gestational age (GA) greater than 20 weeks; regardless of maternal age; presence of comorbidities; sociodemographic profile, and within the study period. Group of Pregnant Women with PE (PE+): pregnant women with blood pressure (BP) ≥ 140/90 mmHg, proteinuria/creatinuria ratio ≥ 30 mg/mol [3]. US-Doppler with uterine artery pulsatility index (UtAPI) > 95^th^ percentile as a predictive factor for PE. Cases diagnosed with HELLP syndrome. Group of Pregnant Women without PE (PE-): pregnant women who do not meet these proposed diagnostic criteria for PE. **Exclusion criteria**: unconfirmed suspected cases, pregnant women transferred to other institutions, SARS-CoV-2 acquired before pregnancy, GA before 20 weeks, multiple pregnancies, pregnant women with PE-like syndrome and HELLP-like syndrome associated with severe COVID-19, to avoid confounding factors with classic HELLP syndrome. However, it was temporary and showed improvement after the regression of viral pneumonia. |  |  |  |  |  |  | **4** |  |
| Data sources/ measurement | 8* | (a) The variables studied included: a) Gestational history: age of the pregnant woman (risk age greater than 34 years: absent or present), parity, usual or high-risk prenatal care; as main comorbidities studied: chronic arterial hypertension, obesity and gestational diabetes; b) Characteristics of COVID-19: tests performed for COVID-19, severity of respiratory symptoms, chest axial computerized tomography (chest CAT) and markers of inflammatory activity; c) Perinatal outcomes: type of delivery, postpartum hemorrhage (PPH), premature rupture of membranes (PROM), histological placental changes, fetal death, prematurity, low birth weight, 5th minute Apgar score, need for hospitalization of the infant newborn in the Neonatal Intensive Care Unit (NICU). In the interpretation of perinatal outcomes, the criteria of the Brazilian Society of Pediatrics; d) Maternal outcomes: ICU admission, duration of COVID-19 symptoms, length of stay in the ICU, need for ventilatory support, plasmapheresis, pronation, maternal corticosteroid therapy and maternal death. The classification of COVID-19 was standardized according to the Brazilian Ministry of Health's Manual of Recommendations for Assistance to Pregnant and Postpartum Women Facing the COVID-19 Pandemic. |  |  |  | 5 |  |  |  |  |
| Bias | 9 | Non-availability of tests to evaluate biomarkers in the laboratories of the participating institutions, for differential diagnosis between HELLP-like and classic HELLP syndrome. The universal screening to SARS-CoV-2, was not performed, and the asymptomatic patients were not included. Type 2 error may have occurred due to the sampling imbalance between the PE+ and PE- groups, which must be interpreted with due caution. |  |  |  | 10 |  |  |  |  |
| Study size | 10 | The sample size was estimated at 93 cases, considering type II error of 10%, magnitude of effect of 10%, significance level of 5%, the prevalence of PE in Brazil of 2% and 12% in pregnant women with COVID-19, with 95% test power |  |  |  | 6 |  |  |  |  |
| Quantitative variables | 11 | Student’s t-test was used to estimate the difference between continuous variables with symmetric distribution and Mann-Whitney test was used for asymmetrical distribution. Fisher's exact test and Pearson's chi-square test were used for categorical variables. Relative risk (RR) was used for univariate analysis of risks associated with maternal outcomes. The multivariate logistic regression model was applied to estimate the main gestational and disease severity factors predictive for PE and main perinatal and maternal outcomes of PE. In the multivariate logistic regression model, the variable PE was considered as a dichotomous response variable |  |  |  | 6 |  |  |  |  |
| Statistical methods | 12 | (*a*) Student’s t-test was used to estimate the difference between continuous variables with symmetric distribution and Mann-Whitney test was used for asymmetrical distribution. Fisher's exact test and Pearson's chi-square test were used for categorical variables. Relative risk (RR) was used for univariate analysis of risks associated with maternal outcomes. The multivariate logistic regression model was applied to estimate the main gestational and disease severity factors predictive for PE and main perinatal and maternal outcomes of PE. In the multivariate logistic regression model, the variable PE was considered as a dichotomous response variable |  |  |  | 6 |  |  |  |  |
|  |  | (*b*) not applicable |  |  |  |  |  |  |  |  |
|  |  | (*c*) was addressed to exclusion criteria |  |  |  | 5 |  |  |  |  |
|  |  | (*d*) was addressed to exclusion criteria |  |  |  | 5 |  |  |  |  |
|  |  | (*e*) not applicable |  |  |  |  |  |  |  |  |
| Results | | |  |  |  |  |  |  |  |  |
| Participants | 13* | (a) gestational history, was observed no difference between PE+ and PE- groups with respect to maternal age (28.6+8.0 *vs* 31.1+6.3 years, p=0.23). Variables such as nulliparity and high risk prenatal were about 40% higher in PE+ group, not statistically significant, but the frequency of comorbidities was significantly higher in the PE+ group (63.6% *vs* 31.5%, p=0.03). The main comorbidities of PE**-** group were diabetes and obesity, while diabetes, obesity, and hypertension were significantly higher in the PE+ group |  |  |  | 7 |  |  |  |  |
|  |  | (b) from 142 patients treated in the emergency rooms of both institutions were selected with confirmed diagnoses of Covid-19 or with a clinical picture highly suggestive of the disease (83 from CHC/UFPR/EBSERH and 59 from CHNSG). Two CHNSG patients refused to participate in the research and two, who had Covid-19 before pregnancy, were excluded. Data were collected from 138 pregnant women infected or suspected of having SARS-CoV-2, all hospitalized. From CHC/UFPR/EBSERH, of a total of 83 pregnant women admitted, 24 were excluded: 11 transferred to other institutions, nine due to unconfirmed RT-PCR tests, two with twin pregnancy and two with Like-PE syndrome or HELLP-like syndrome , therefore leaving 59 participants. From the CHNSG, of the total of 55 pregnant women admitted, 14 were excluded: eight due to unconfirmed RT-PCR tests, two transferred to other institutions, two with twin pregnancies and two due to Like-PE syndromes or HELLP-like syndrome, described as inflammatory syndromes similar to classic HELLP syndrome, temporary, associated with severe Covid-19, which showed clinical and laboratory improvement after the end of viral pneumonia. Therefore, 41 participants from this institution were included. Thus, 100 pregnant women with Covid-19 and their 100 respective newborns were included. **Figure 1** |  |  |  | 6 |  |  |  |  |
|  |  | (c) We use a Flowchart according to PRISMA (2020) |  |  |  | 6 |  |  |  |  |
| Descriptive data | 14* | 1. Inclusion criteria: hospitalized pregnant women with positive tests for SARS-CoV-2, through nasopharyngeal real time polymerase chain reaction (RT-PCR), rapid antigen test, and IgM and IgG serology; with gestational age (GA) greater than 20 weeks; regardless of maternal age; presence of comorbidities; sociodemographic profile, and within the study period. Group of Pregnant Women with PE (PE+): pregnant women with blood pressure (BP) ≥ 140/90 mmHg, proteinuria/creatinuria ratio ≥ 30 mg/mol [3]. US-Doppler with uterine artery pulsatility index (UtAPI) > 95th percentile as a predictive factor for PE. Cases diagnosed with HELLP syndrome. Group of Pregnant Women without PE (PE-): pregnant women who do not meet these proposed diagnostic criteria for PE. Exclusion criteria: unconfirmed suspected cases, pregnant women transferred to other institutions, SARS-CoV-2 acquired before pregnancy, GA before 20 weeks, multiple pregnancies, **pregnant women with PE-like syndrome and HELLP-like syndrome associated with severe COVID-19, to avoid confounding factors with classic HELLP syndrome**. However, it was temporary and showed improvement after the regression of viral pneumonia. 2. During the research, the data collection tools used were the software Tasy, the Management Application for University Hospitals, and the Hospital Information System. Microsoft Excel spreadsheets were used to record the data. The variables studied included: a) Gestational history: age of the pregnant woman (risk age greater than 34 years: absent or present), parity, usual or high-risk prenatal care; as main comorbidities studied: chronic arterial hypertension, obesity and gestational diabetes; b) Characteristics of COVID-19: tests performed for COVID-19, severity of respiratory symptoms, chest axial computerized tomography (chest CAT) and markers of inflammatory activity; c) Perinatal outcomes: type of delivery, postpartum hemorrhage (PPH), premature rupture of membranes (PROM), histological placental changes, fetal death, prematurity, low birth weight, 5th minute Apgar score, need for hospitalization of the infant newborn in the Neonatal Intensive Care Unit (NICU). In the interpretation of perinatal outcomes, the criteria of the Brazilian Society of Pediatrics [13]; d) Maternal outcomes: ICU admission, duration of COVID-19 symptoms, length of stay in the ICU, need for ventilatory support, plasmapheresis, pronation, maternal corticosteroid therapy and maternal death. **Page 4** |  | 1. 4 | 1. 4 | 1. 444 | 1. 4 | 1. 4444444 | 1. 444 | 1. 4 |
|  |  |  |  |  |  |  |  |  |  |  |
|  |  | (c) 142 patients treated in the emergency rooms of both institutions were selected with confirmed diagnoses of Covid-19 or with a clinical picture highly suggestive of the disease (83 from CHC/UFPR/EBSERH and 59 from CHNSG). Two CHNSG patients refused to participate in the research and two, who had Covid-19 before pregnancy, were excluded. Data were collected from 138 pregnant women infected or suspected of having SARS-CoV-2, all hospitalized. From CHC/UFPR/EBSERH, of a total of 83 pregnant women admitted, 24 were excluded: 11 transferred to other institutions, nine due to unconfirmed RT-PCR tests, two with twin pregnancy and two with Like-PE syndrome or HELLP-like syndrome , therefore leaving 59 participants. From the CHNSG, of the total of 55 pregnant women admitted, 14 were excluded: eight due to unconfirmed RT-PCR tests, two transferred to other institutions, two with twin pregnancies and two due to Like-PE syndromes or HELLP-like syndrome, described as inflammatory syndromes similar to classic HELLP syndrome, temporary, associated with severe Covid-19, which showed clinical and laboratory improvement after the end of viral pneumonia. Therefore, 41 participants from this institution were included. Thus, 100 pregnant women with Covid-19 and their 100 respective newborns were included. Figure 1 |  |  | 6 |  |  |  |  |  |
|  |  | (c) The prevalence of preeclampsia was 11%. Severe COVID-19 was the main risk factor for preeclampsia (OR = 8.18 [CI 1.53-43.52]), as well as fetal growth restriction was the main perinatal outcome (OR=8.90 [CI 1.52-38.4]). Comorbidities were more frequent in the PE+ group (63.6% *vs* 31.5%, p=0.03), as well as prematurity (81.8% *vs* 41.6%, p=0.02), low birth weight (63.6% *vs* 24.7%, p=0.01), and the need for neonatal intensive care admission of the newborn (63.6% *vs* 27.0%, p=0.03). Pregnant women with PE had twice as long a length of stay in the intensive care unit (RR = 2,35 [CI 1,34-4,14]). Although maternal mortality was more frequent among pregnant women with PE, it was not statistically significant. |  |  | 2 |  |  |  |  |  |
| Outcome data | 15* | Severe COVID-19 was the main risk factor for preeclampsia (OR = 8.18 [CI 1.53-43.52]), as well as fetal growth restriction was the main perinatal outcome (OR=8.90 [CI 1.52-38.4]). Comorbidities were more frequent in the PE+ group (63.6% vs 31.5%, p=0.03), as well as prematurity (81.8% vs 41.6%, p=0.02), low birth weight (63.6% vs 24.7%, p=0.01), and the need for neonatal intensive care admission of the newborn (63.6% vs 27.0%, p=0.03). Pregnant women with PE had twice as long a length of stay in the intensive care unit (RR = 2,35 [CI 1,34-4,14]). The prevalence of preeclampsia was 11%. |  |  | 7 |  |  |  |  |  |
| Main results | 16 | (*a*) unadjusted estimates not applicable and confounder-adjusted estimates were not included |  |  |  |  |  |  |  |  |
|  |  | (*b*) gestational history, was observed no difference between PE+ and PE- groups with respect to maternal age (28.6+8.0 *vs* 31.1+6.3 years, p=0.23). Variables such as nulliparity and high risk prenatal were about 40% higher in PE+ group, not statistically significant, but the frequency of comorbidities was significantly higher in the PE+ group (63.6% *vs* 31.5%, p=0.03). |  |  | 6 |  |  |  |  |  |
|  |  | (*c*) Among maternal outcomes, the length of stay in the ICU was longer in the PE+ group (p=0.04) which occurred on average at 9.7+2.5 days of stay. And no differences were observed in the frequency of maternal deaths (p=0.29), one in the PE+ group and two in the PE- group. The frequency of need for ICU admission and pronation were also higher in PE+ group (p=0.06 and p=0.08). In the univariate analysis it indicated a two times higher risk for ICU admission and longer ICU stays in PE+ group (RR = 2,35 [CI 1,34-4,14] p=0.04) **Page 7** |  |  | 7 | 77 | 7 |  |  |  |
| Other analyses | 17 | Relative risk (RR) was used for univariate analysis of risks associated with maternal outcomes. The multivariate logistic regression model was applied to estimate the main gestational and disease severity factors predictive for PE and main perinatal and maternal outcomes of PE. In the multivariate logistic regression model, the variable PE was considered as a dichotomous response variable. As a measure of effect, was estimated the relative risk (RR) for all variables studied and its 95% confidence interval (CI). The p value <0.05 is an indicator of statistical significance. |  |  |  | 6 |  |  |  |  |
| Discussion | | |  |  |  |  |  |  |  |  |
| Key results | 18 | The prevalence of preeclampsia was 11%. Severe COVID-19 was the main risk factor for preeclampsia (OR = 8.18 [CI 1.53-43.52]), as well as fetal growth restriction was the main perinatal outcome (OR=8.90 [CI 1.52-38.4]). Comorbidities were more frequent in the PE+ group (63.6% *vs* 31.5%, p=0.03), as well as prematurity (81.8% *vs* 41.6%, p=0.02), low birth weight (63.6% *vs* 24.7%, p=0.01), and the need for neonatal intensive care admission of the newborn (63.6% *vs* 27.0%, p=0.03). Pregnant women with PE had twice as long a length of stay in the intensive care unit (RR = 2,35 [CI 1,34-4,14]). |  |  |  | 2 |  |  |  |  |
| Limitations | 19 | Discuss limitations of the study: the non-availability of tests to evaluate biomarkers in the laboratories of the participating institutions, for differential diagnosis between HELLP-like and classic HELLP syndrome. The universal screening to SARS-CoV-2, was not performed, and the asymptomatic patients were not included. Type 2 error may have occurred due to the sampling imbalance between the PE+ and PE- groups, which must be interpreted with due caution. |  |  |  | 10 |  |  |  |  |
| Interpretation | 20 | When COVID-19 is associated with pregnancy, it increases the prevalence of PE by 11%. This is about 3.7 times higher than data from the pre-pandemic period, in the same institutions. Severe COVID-19 was the main predictive factor for PE, increasing its risk by eight times, and fetal growth restriction was the main perinatal outcome of PE, with the same magnitude. Pregnant women with PE were two times more likely to require ICU admission, and the length of stay in the ICU was longer in PE+ group. Associated comorbidities were more frequent in the PE+ group. Maternal mortality was more frequent among pregnant women with PE, but there was no significant difference between groups. The placental morphological changes were 30% higher in the PE+ group. Prematurity, low birth weight and need for NICU admission were also more prevalent in the PE+ group. **Page 11** |  |  | 111 |  |  |  |  |  |
| Generalisability | 21 | The most important and practical information that can be extracted from this study is that when we are faced with a pregnant woman with COVID-19, the chance of association with PE is greater. If COVID-19 is severe or the pregnant woman shows comorbidities this chance increases to much. On the other hand, when in the pregnant woman with COVID-19 the PE is installing the chance of the severity of the case and the necessity of ICU has increased |  |  |  | 10 |  |  |  |  |
| Other information | | |  |  |  |  |  |  |  |  |
| Funding | 22 | This work may be partially financed to pay off the APC fee for this journal, and will be paid by the FUNPAR Foundation - Ministry of Education - Brazil - Federal University of Paraná in up to USD 1,000 |  |  |  |  |  |  |  |  |

*Give information separately for exposed and unexposed groups.

**Note:** An Explanation and Elaboration article discusses each checklist item and gives methodological background and published examples of transparent reporting. The STROBE checklist is best used in conjunction with this article (freely available on the Web sites of PLoS Medicine at http://www.plosmedicine.org/, Annals of Internal Medicine at http://www.annals.org/, and Epidemiology at http://www.epidem.com/). Information on the STROBE Initiative is available at http://www.strobe-statement.org.
